# Evolutionary Path and Innovative Development of Pharmaceutical Industrial Cluster—A Case Study of Shijiazhuang, China

**DOI:** 10.3390/ijerph19052928

**Published:** 2022-03-02

**Authors:** Liping Fu, Fan Wu, Shan Zhang

**Affiliations:** College of Management and Economics, Tianjin University, Tianjin 300072, China; lpf3688@126.com (L.F.); zslois@163.com (S.Z.)

**Keywords:** innovative development, evolutionary path, pharmaceutical industrial cluster, case study

## Abstract

The innovation and development practices of the pharmaceutical industry are of great importance for the continual enhancement of public health. Industrial clusters are one of the important means by which the pharmaceutical industry can be transformed and developed, but there is a lack of research on the evolutionary path and development innovation of pharmaceutical industrial clusters. As a country with a major pharmaceutical industry, the transformation and development path of traditional pharmaceutical industrial clusters in China has important reference value for the sustainable development of the global pharmaceutical industry. Through an analysis of the evolution of traditional pharmaceutical industrial clusters in Shijiazhuang, this study explores the law of the dynamic evolution of pharmaceutical industrial clusters and the associated mechanisms. Specifically, we conclude that the evolutionary path of traditional pharmaceutical industrial clusters can be divided into the government-led pharmaceutical industrial cluster start-up stage, the government-guided pharmaceutical industrial cluster development stage, and the government-served pharmaceutical industrial cluster transformation stage. The operating mechanisms include a self-organization mechanism, an innovation-driven mechanism, and an outward associated mechanism, each of which plays different roles in the different stages of the dynamic evolution of the cluster, and the synergistic operation of the three mechanisms forms an important basis for the evolution of pharmaceutical industrial clusters. We found that the innovation development of traditional pharmaceutical industrial clusters is influenced by the synergy of the self-organization, innovation-driven, and outward associated mechanisms. The self-organization mechanism is a necessary condition for pharmaceutical industrial clusters to realize the transition from disorder to order. The innovation-driven mechanism is the core driving force for the innovative development of pharmaceutical industrial clusters. Finally, the outward associated mechanism is the main means by which pharmaceutical industrial clusters integrate into the global value chain.

## 1. Introduction

As a typical technology-intensive industry, the pharmaceutical industry is one of the fastest developing industries in the world. The pharmaceutical and biopharmaceutical sectors have seen significant changes in their operating model and footprint over the past couple of decades [1]. In recent years, as global trade frictions continue to intensify, anti-globalization development has become prevalent. Due to these factors, along with the impacts of the COVID-19 pandemic, the innovation and development of the global industrial ecology have been greatly impacted. The implementation of Industry 4.0 has also played an increasingly significant role in the modernization of pharmaceutical industries [2]. In the complex and changeable global industry pattern, how to innovate and develop the pharmaceutical industry, according to its own characteristics, is very important.

China is an important participant in the development of the global pharmaceutical industry. In the context of the continuous adjustment of the global industrial structure, China’s economic development has entered a new normal period. There is an urgent need to create new driving forces for economic and social development, through new technological revolutions and industrial transformations. Industrial clusters are clusters of companies or institutions that are geographically close to each other, which belong to or are associated with a particular industry, are inter-related and complement each other [3], and comprise an important form of organization for the development of industrial change. We rely on new technologies, industries, and models to replace the traditional business models based on resource consumption, and seek to replace old growth drivers with new ones. In this process, the development of pharmaceutical industrial clusters has become an important link in the context of economic growth.

At present, the development of traditional pharmaceutical industrial clusters in China is facing increasingly prominent problems, in terms of rising labor costs, the global economic downturn, and the strengthening of resource and environmental constraints [4]. At the same time, the “status quo bias” in industry chain channels and sales links also restricts the expansion of pharmaceutical industrial clusters to overseas markets to a large extent. The following questions arise: How do pharmaceutical industrial clusters evolve and develop innovation? Which subjects play a key role in the formation and evolution of the pharmaceutical industry? What are the possible mechanisms of action? Analyzing the law of the development of pharmaceutical industrial clusters is of great significance to the development and innovation of the pharmaceutical industry under the background of anti-globalization, and is also meaningful for the healthy and sustainable development of the public health industry.

The life cycle of industrial clusters includes aspects from the emergence of industrial clusters to the decline of the whole course, along with the development of industrial clusters generally through the germination period, the formation period, the growth period, the maturity period, and the decline period [5]. Academic research on the innovative development of industrial clusters began with Marshall’s “industrial zone” theory and Weber’s “industrial location” theory, which explored external economies of scale [6]. To date, scholars have mainly analyzed the mechanism of innovation development of industrial clusters based on Porter’s diamond model, new industrial zone theory, and global value chain theory. Based on the diamond model, many factors affecting cluster development have been proposed, including human resources, trade costs and technological differences between industries, service level, and the strength of industry chain governance; consequently, it is believed that industry linkages and self-reinforcing mechanisms influence the innovative development of clusters [7]. The new industrial location theory emphasizes the role of the formation of regional innovation networks in promoting the innovative development of industrial clusters, and that the subject, function, and environmental elements of innovation contribute to the competitiveness of clusters [8]. Global value chain theory focuses on the innovative development of industrial clusters through strengthening external linkages, based on process, product, functional, and chain upgrading, among other aspects [9].

The pharmaceutical industry differs from any other technology-intensive industry due to its high complexity and tacit-knowledge-intensive innovation environment, as well as the inherent difficulties and non-linear innovation processes [10]. In research on the innovation development of specific industrial clusters, the formation, growth, and sustainable development of clusters are taken as the main object. Scholars have concluded that the mechanisms of innovation development in industrial clusters include, among others, technological capabilities, innovation capabilities, and external linkages, and it is believed that the innovative development of industrial clusters requires the synergy of endogenous and exogenous dynamics [11,12]. Collaborative innovation is an innovation organization model with a large span of integration among enterprises, governments, knowledge-producing institutions, intermediaries, and users, in order to achieve major scientific and technological innovation with value-added knowledge as the core [13]. In the process of collaborative innovation development, industrial clusters can fully mobilize endogenous and exogenous dynamics to generate regional scale effects, and exogenous resources in the perspective of global value chain serve as exogenous factors promoting the flow of knowledge within the system, thus promoting industrial collaborative innovation [14]. However, market failures also occur, in response to which, the relevant governmental bodies need to intervene and introduce relevant industrial policies to deal with the shortcomings of the free market.

After experiencing the limited government stage, as well as modern state interventionism, neoliberalism, and mixed economy, mainstream economic theory has reached a basic consensus on the relationship between the government and the market: the market is an effective means of resource allocation, and appropriate government intervention is necessary when the market fails. In the process of industrial cluster development, local governments use administrative means to intervene in microeconomic activities [15,16], play an important role in intervention and service, and comprise the most important actor in technological innovation in industrial clusters [17,18]. In the evolutionary upgrading process of industrial clusters, local governments have the responsibility to provide public goods with externalities, such as technology exchange platforms and R & D support [19]. The behavior and role of local governments change dynamically, according to the characteristics of industries or clusters, and this process requires both an “effective market” and an “active government” [20,21].

As a complex industrial system, the pharmaceutical industry has its own development characteristics, development rules, and innovative development mechanisms. The law of traditional industrial development cannot fully explain the growth and development of the pharmaceutical industry. The existing literature lacks a systematic analysis of the internal mechanism of innovation and development of pharmaceutical industrial clusters from a dynamic perspective linking the two dimensions of global and local development.

Based on the practical needs and theoretical gap mentioned above, in this paper, we take the pharmaceutical industrial cluster selected by the Ministry of Science and Technology of the People’s Republic of China as the research object. Using a longitudinal case study, we analyze the internal logic of the evolution of the traditional medicine industry cluster to the innovation cluster, as well as the innovative development process of pharmaceutical industrial clusters, and then explore the path and internal mechanism of pharmaceutical industrial cluster innovative development.

## 2. Materials and Methods

### 2.1. Method Selection

In this study, we mainly explore the mechanisms of development and innovation for traditional pharmaceutical industrial clusters, considering “how” and “why” questions, which are appropriate for case study methods. At the same time, compared with a multi-case study, a single case study can dig deeper into the considered phenomenon [22], thus enhancing the persuasiveness of the theory [23]. In particular, a single case study has obvious advantages for in-depth investigation of new phenomena and dynamic processes, making it conducive to reflecting the changes of the cases studied at different stages of development, in order to better examine the issues raised in the research framework [24,25]. Therefore, we adopted the exploratory single case study method to try to open the black box of the evolution and upgrading of pharmaceutical industrial clusters, thus further deepening understanding of the sustainable development of the pharmaceutical industry.

### 2.2. Case Selection and Data Collection

According to the purpose of this study, the following points were considered when selecting the case cluster: (1) selecting clusters that have been established for more than 35 years, in order to ensure that the cluster evolved throughout its life cycle, and to improve the typicality of the case; (2) the innovation industrial cluster pilot established by the state was selected to enhance the representativeness of the case; and (3) the selected industrial cluster and the research members were in the same geographical region (Beijing–Tianjin–Hebei), and both parties have good relationships, which is conducive to regular field research. Based on the above conditions, we finally chose the Shijiazhuang pharmaceutical excipients and preparations industrial cluster as the case object.

**Figure 1:**
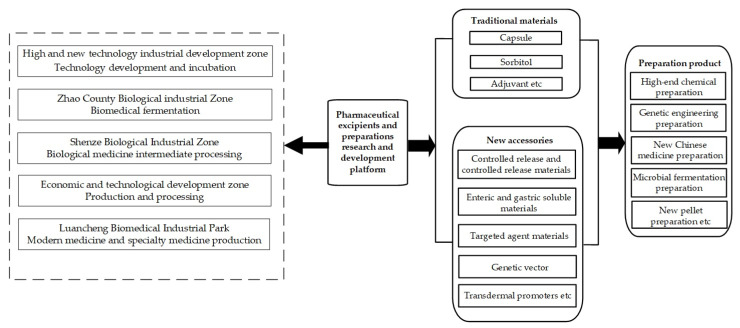
Shijiazhuang pharmaceutical excipients and preparations industrial cluster.

The Shijiazhuang pharmaceutical excipients and preparations industrial cluster selected for this study is an important global production base for vitamin and antibiotic raw materials and preparations, was in the first batch of national bio-industry bases, and is one of the largest pharmaceutical industry bases and modern Chinese medicine production bases in China. The formation of the cluster can be traced back to the anti-Japanese war period, and its development has gone through a budding period, a creation period, and a rapid development period, and is currently in a period of transformation and development. A new pharmaceutical pattern featuring biopharmaceuticals, green Chinese medicine, new preparations, and plant extract products has formed, as well as five zones with different functions. The industrial cluster composition is shown in Figure 1.

The data processing conducted for this paper mainly included qualitative analysis of the collected data, and summary processing of all text data. Relevant data were analyzed by different researchers, in order to ensure the reliability of the study. In accordance with the relevant order of case analysis, the original data were qualitatively pre-processed. The development stages were divided according to the evolution law of the growth and innovation of pharmaceutical industrial cluster, mainly identifying key events in the development process of industrial clusters, and obtaining relevant explanations from raw data. The details of data collection and data composition are shown in Table 1.

## 3. Evolution Path of the Traditional Pharmaceutical Industrial Clusters to Innovation Clusters

### 3.1. Initial Stage of Pharmaceutical Industrial Cluster (Before 2003)

Historically, the most basic condition for the sprouting and formation of the industry was the material and cultural needs of the population, and the shortage of medical supplies during the war period laid the foundation for the development of Shijiazhuang’s pharmaceutical industry. Capital support and a stable supply of resources allowed for the formation of the industry to provide basic protection; after the founding of New China, the Soviet Union, to assist in the construction of pharmaceutical plants, as well as the country’s development of medical and health care, invested a large amount of money in the pharmaceutical industrial cluster in Shijiazhuang. The government’s industrial policy has contributed to the sustained and stable development of Shijiazhuang’s pharmaceutical industry, along with the successive establishment of the Shijiazhuang National High-tech Industrial Development Zone, and the Shijiazhuang Economic and Technological Development Zone. The implementation of preferential policies for the development of the pharmaceutical industry has attracted a large number of investments, after which a large number of pharmaceutical enterprises gathered in the development zone, and the scale of industrial development has begun to bear fruit.

The coding strategy in this paper is mainly based on the method proposed by Patton [26]. The raw data are pre-processed, secondary codes are extracted, the codes are classified and arranged, sub-concepts are abstracted, then sub-concepts are classified, and finally, the main concepts are defined. Table 2 was coded by collating the transcripts of interviews involving the start-up stage of the pharmaceutical cluster. We found that this stage of cluster development comprised a government-led agglomeration process. Initial cluster formation was due to historical contingent factors, whereas initial advantages were magnified by path dependence, and incremental payoffs of scale manifested themselves, resulting in a geographical lock-in effect [27]. Resource integration within the cluster and governmental management formed the core of development; the self-organization mechanism was the focus of development at this stage, and the roles of the innovation-driven mechanism and outward associated mechanism were also mainly reflected through relevant government policies [28]. In the process of government-led cluster development, the degree of interaction within the cluster is low, and the connection with the outside stays more at the stage of introduction and absorption, mainly being engaged in the processing and production of Active Pharmaceutical Ingredients (APIs) and other aspects of the work.

### 3.2. Development Stages of Pharmaceutical Industrial Clusters (2003–2013)

Under the influence of the market economy, the degree of government-led industrial agglomeration development is always limited, and technological progress is the core force of industrial change and evolution [29]. According to the development of the industry, enterprises within the cluster began to seek innovative cooperation, with North China Pharmaceutical Company (NCPC) and China ShiYao Pharmaceutical Group Company (CSPC) as the leading enterprises within the cluster. They gradually began to establish relevant scientific research institutions, establish cooperation with universities, and introduced a high volume of professional talent. The formation of drug research and development, API manufacturing, and finished drug sales led to the whole industry chain model of finished drug sales.

In 2013, the Shijiazhuang pharmaceutical excipients and preparations industrial cluster was included in the national pilot innovative industrial cluster by the Ministry of Science and Technology of the People’s Republic of China, where the development mode of the cluster gradually began to change from the traditional agglomeration of enterprises to the construction of a cluster innovation network. Table 3 was coded by collating the transcripts of interviews involving the development stage of the pharmaceutical cluster. We found that, at this stage of cluster development, the role of the government gradually diminished, changing from a dominant position to a guiding position. The innovation cooperation network within the cluster was gradually formed, and the independent R & D activities of enterprises, universities, and research institutions were the core driving force for the development of the cluster [30].

### 3.3. Innovation Stages of Pharmaceutical Industrial Clusters (Since 2013)

With China playing an increasingly important role in global economic development, clusters have gradually integrated into the global pharmaceutical industry chain, based on internal collaborative innovation and taking advantage of API production, bringing new development opportunities for traditional clusters whose development has entered a bottleneck. When the innovation network within the cluster is basically built, the cluster faces the pressure of transformation and upgrading, and the pharmaceutical companies within the cluster start to strengthen their innovation investment, and actively apply for certification in international regulated markets. The pharmaceutical enterprises in the cluster have gradually strengthened their independent research and development from raw material enterprises in the international division of labor, and have finally realized the international circulation of drugs, thus basically covering the whole industrial chain of production. Table 4 was coded by collating the transcripts of interviews involving the innovation stage of the pharmaceutical cluster. It was found that, at this stage, the government no longer needs to guide the development of industrial clusters, and the government is more interested in providing some macro-level services to provide a good environment for the interaction of various organizations within the industrial clusters for innovation development. Orderly interactions between the self-organization mechanism, innovation-driven mechanism, and outward linkage mechanism are an important feature in transformation and upgrading development. The focus of innovation cluster development has also shifted from domestic industrial competition to international division of labor cooperation, climbing to the top end of the global value chain production division of labor, acquiring more added value, and enhancing the core competitiveness of cluster development.

## 4. Mechanism of Pharmaceutical Industrial Cluster Innovation Evolutionary

### 4.1. Self-Organizing Mechanism

**Figure 2:**
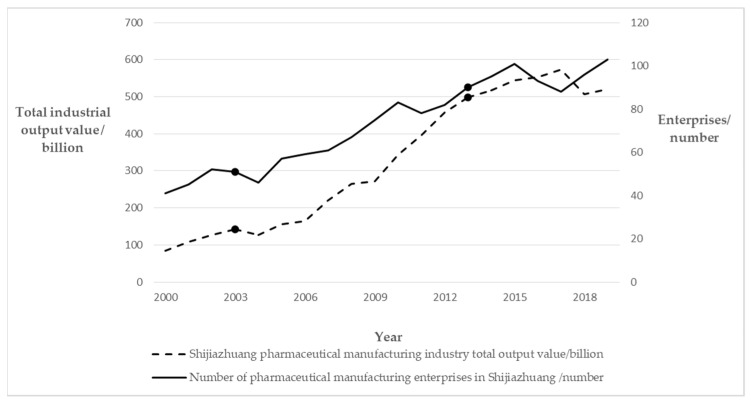
Shijiazhuang pharmaceutical manufacturing enterprises and total industrial output value. (Data source: Shijiazhuang Statistical Yearbook 2001–2020).

One of the most important mechanisms for the evolution of innovation in the pharmaceutical industrial cluster is the self-organization mechanism. In economic activities, the organizations and elements within the cluster are both open and closed, both competitive and cooperative, and contribute to the continuous differentiation and integration of the whole system through their interaction, realizing the continuous development from equilibrium to disruption of equilibrium, and then to a new equilibrium [31]. Figure 2 shows the growth of enterprises and the growth of total industrial output value of the pharmaceutical manufacturing industry in Shijiazhuang. It can be seen that with the increasing self-organization ability of the cluster, the pharmaceutical manufacturing industry achieved rapid development between 2003 and 2013, and the number of enterprises and total industrial output value also showed a fluctuating upward trend after 2013.

In the process of pharmaceutical industrial cluster management innovation, a complete R & D–manufacturing–wholesale and retail–industrial chain is formed in the cluster, and the cluster’s main body reduces transaction costs, and promotes effective resource management through the vertical division of labor and cooperative synergy with the upstream and downstream of the industrial chain. Through the vertical division of labor and cooperation and synergy with the upstream and downstream of the industry chain, related industries are driven to carry out progressive innovation, thus forming innovation synergy [32]. In the horizontal network relationship, the synergy between competition and cooperation among similar enterprises, as well as that between enterprises and local governments, universities, and research institutions in R & D, accelerates the flow of knowledge and various resources between organizations [33].

The Shijiazhuang pharmaceutical industrial cluster consists of five different functional zones, which respectively hold their own professional advantages: An Economic and Technological Development Zone, with a focus on production and processing; a High-tech Development Zone, with a focus on technology development and incubation; Zhao County Bio-Industrial Zone, with a focus on the biopharmaceutical fermentation industry; Luancheng Bio-Pharmaceutical Industrial Park, with a focus on modern Chinese medicine and specialty drug production; and Shenze Bio-Industrial Zone, with a focus on the processing of biopharmaceutical intermediates. Enterprises within the cluster have established extensive links with multiple universities, hospitals, research institutes, and so on. Ultimately, the synergy between the vertical and horizontal dimensions makes the value created among cluster subjects far greater than the value of innovation with respect to enterprises alone, promoting the transformation and upgrading of traditional pharmaceutical industrial clusters.

### 4.2. Innovation-Driven Mechanism

Building and improving the cluster innovation network is the key to the evolution of pharmaceutical industrial clusters. First, the allocation of pharmaceutical resource elements should be optimized. According to Schumpeter, innovation is the introduction of never-before-seen combinations of production factors and production conditions into the production system, the upgrading of production factor resources or the optimal combination of production factors, the integration of innovation resources in the region, the formation of regional innovation synergy, and the improvement of production and output efficiency of industrial clusters, thus promoting the innovative development of industrial clusters [34,35]. The Shijiazhuang pharmaceutical excipients and preparations industrial cluster concentrates more than 95% of the pharmaceutical science and technology innovation elements in Hebei Province. Several innovation platforms and the only State Key Laboratory of “New Drug Formulation and Excipients” in China were built, which not only includes the Institute of Formulation Technology, the Institute of Synthesis Technology, and so on, but also has a supporting synthesis pilot and formulation multi-functional pilot R & D platform that meets international certification standards. It is a first-class incubation base for new drugs in China, with strong innovation capability in new formulation research, analysis and testing, pharmacological research, and so on.

Second, the interaction and synergy of cluster subjects should be promoted. The pharmaceutical industrial cluster is composed of a large number of enterprises, with division of labor and cooperation relations between various institutions, organizations, and other actors related to development, concentrated in a certain area of the pharmaceutical industry [36]. Due to the plurality of subjects, innovation involves not only the behavior of a particular enterprise, but also forms an effective network through the subjects of the innovation system (e.g., enterprises, research institutions, universities, intermediary services, and local governments) to form an innovation system [37,38]. The establishment of several strategic alliances has accelerated the innovation development of the cluster, and several integrated innovation systems have been established in the cluster [39,40], such as the Antibiotics Industrial Technology Innovation Strategic Alliance, the China Drug Technology Innovation and Industrialization Strategic Alliance, and the Innovative Drug Development Industry University Research Alliance, among others. Rich innovation resources provide strong support for cluster technology innovation.

**Figure 3:**
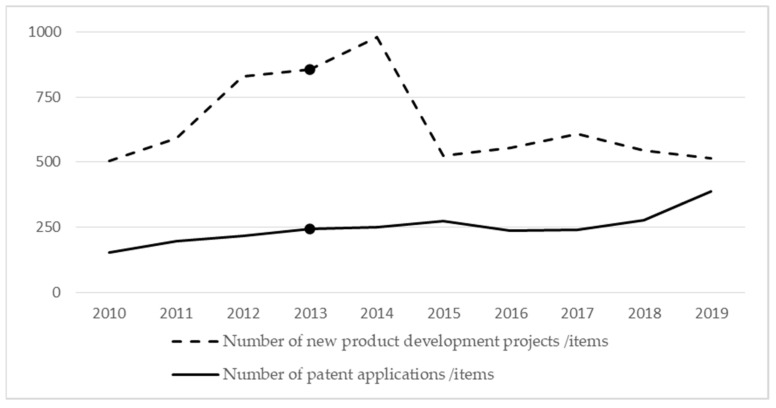
Shijiazhuang pharmaceutical manufacturing new product development projects and patent applications. (Data source: Shijiazhuang Statistical Yearbook 2001–2020).

Third, the regional innovation environment should be integrated. The pharmaceutical industrial cluster, as a regionally rooted organizational system, features innovative development that is bound to occur in close contact with the regional environment. The regional innovation environment is transformed into the intrinsic motivation of cluster innovation through induction and stimulation [41]. When the industrial clusters and the external environment are coordinated and mutually beneficial, they can obtain more support from innovation factors, maintain and enhance the competitive advantage of the clusters, and achieve upgraded development [42]. With the continuous and in-depth promotion of the Beijing–Tianjin–Hebei synergistic development strategy, the Shijiazhuang pharmaceutical excipients and preparations industrial cluster is expected to experience large increases in domestic patent authorization, foreign patent authorization, and the number of standards developed or participated in the development of the number of well-known products at the provincial level (brand). Figure 3 shows the new product development projects and patent applications in the pharmaceutical manufacturing industry in Shijiazhuang from 2010–2019. Before 2013 was the period of accelerated development of the cluster, and the number of new product development projects and patent applications grew rapidly. After 2013, with the continuous improvement of the cluster’s innovation capacity, more attention was paid to the improvement of quality in new product development projects and patent applications, rather than simply pursuing the increase of quantity.

### 4.3. Outward Associated Mechanism

Vertical linkages between cluster firms and foreign core value chain players play an important role in innovation for labor-intensive clusters, and the outward linkage mechanism is an important mechanism for the innovative development of pharmaceutical industrial clusters [43]. The interaction between clusters and the external environment can facilitate the transfer of new knowledge, the development of new technologies, the input of new information, and the establishment of global communication channels [44]. In the modern globalized economy, the establishment of global communication channels can provide new market and technological information for enterprises in the cluster, introduce new innovation paradigms to the cluster, and promote the transformation and processing of knowledge within the cluster, thus improving the utilization of knowledge within the cluster [45]. In the connection and communication with the external environment, cluster enterprises may generate new and clearer innovation goals, and innovate through the processing and transformation of knowledge on the basis of their original views, ultimately improving the competitiveness and adaptability of the cluster as a whole [46,47]. At the same time, various standards in the global value chain provide a new international perspective for the construction and improvement of innovation networks [48,49], and promote the convergence of local innovation networks with international innovation networks, in turn, injecting vitality into the development of local industrial clusters. The export of APIs from the Shijiazhuang pharmaceutical excipients and preparations industrial cluster causes the cluster to gradually get out of the local area and come into contact with new knowledge, technology, and information in the international market, providing strong support for local enterprises to carry out innovative R & D through the reprocessing and transformation of knowledge within the cluster.

In recent years, with the intensification of competition among countries to attract foreign investment, the role of traditional governmental preferential policies has gradually decreased, and the openness of the region is the first factor for the introduction of foreign investment [50]. The introduction of foreign enterprises—especially the entry of leading enterprises—can enhance the visibility and influence of the cluster in the industry, triggering downstream supporting enterprises to invest in the local area, and take advantage of their knowledge in technology, mechanisms, and ideas to stimulate local innovation activities, which will gradually expand the scale of the industry, and enhance the cluster effect. Although the introduction of foreign capital can be driven by outward linkages, the manner and degree of integration between local and foreign enterprises has to be considered in the specific operation [51].

### 4.4. Government Role Mechanism

**Figure 4:**
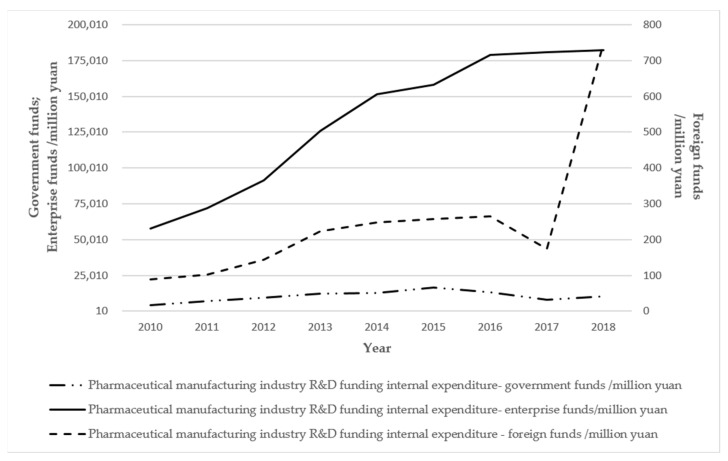
Shijiazhuang pharmaceutical manufacturing industry industrial enterprises above the size of the internal expenditure on R & D funds. (Data source: Shijiazhuang Statistical Yearbook 2001–2020).

Pharmaceutical industrial cluster development, to a certain stage, often involves problems such as a shortage of land indicators, resource constraints, infrastructure mismatching, and so on. The solutions to these problems often do not work when solely relying on the cluster enterprises; the government also needs to step in, according to the different characteristics of the development stage of the cluster, in order to take appropriate measures to address the situation. The government guides the development of the local economy by formulating and improving the policy system [52,53]. The government’s economic behavior mainly refers to the allocation and regulation of production factor resources, using fiscal revenue and fiscal policy to directly or indirectly guide the allocation of resources in the market [54]. Government actions play an important role in regulating the evolutionary stages of industrial cluster evolution and the overall evolutionary rate [55]. The government has issued policies, such as “Policies for Promoting the Development of Biomedical Industry in Shijiazhuang High-tech Zone”, “Several Policies for Promoting the Development of Medical Device Industry in Shijiazhuang High-tech Zone”, and so on, in order to promote the innovative development of cluster biomedicine, and further develop collaboration in the industrial chains driven by leading enterprises and small- and medium-sized enterprises to facilitate innovation. Figure 4 shows the internal expenditure on R & D expenses of industrial enterprises above the scale of the pharmaceutical manufacturing industry in Shijiazhuang from 2010–2018. It can be seen that, as discussed in Section 3, the leading role of the government is gradually diminishing, and tends to provide stable service support for the cluster. Firms’ investment in independent innovation increases year by year, while at the same time, the foreign funding component, which represents the capacity for external linkages, shows an upward trend.

Local governments, as representatives of local public interests, are the main providers of public goods and services, thus playing the role of an effective government in terms of infrastructure, and supporting production services in the process of industrial cluster development. The government promotes the development of the biomedical industry to the high-end of the industrial chain and value chain. For the synergistic development of biopharmaceutical industrial clusters, specific targeted initiatives have been proposed, including supporting the development, production, and marketing of biopharmaceuticals, genetically engineered drugs, and other high-tech biopharmaceuticals; promoting the development and engineering of large-scale cell culture and purification, antibody coupling, serum-free protein-free medium culture, and other biotechnologies; and supporting enterprises to develop and industrialize new antibodies, proteins and peptides, and other biological drugs.

**Figure 5:**
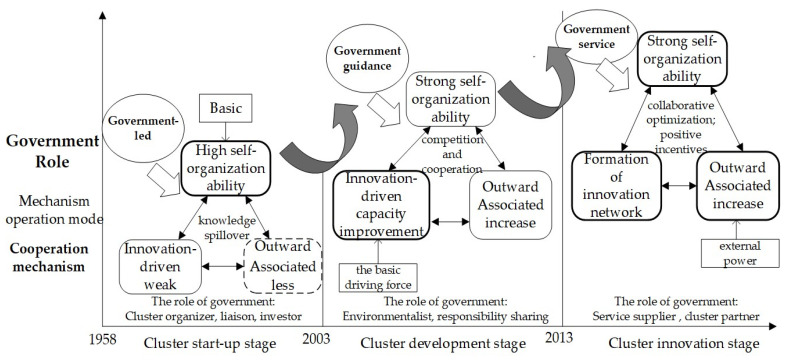
Shijiazhuang pharmaceutical industrial cluster innovation development process.

In this paper, through a case study of the pharmaceutical industry cluster in Shijiazhuang—which was transformed from a traditional industrial cluster into an innovative industrial cluster—we found that the government plays different roles in different stages of industrial cluster development. It is effective for the government to intervene in innovation, and promote the transformation and upgrading of industrial clusters based on their self-organization ability, innovation drive (as the fundamental driving force), and outward linkage (as the external driving force). At the initial stage of industrial cluster development, the self-organizing ability within the cluster is strong, the innovation-driven ability is weak, and there is basically no outward correlation. Based on the stage mechanism of cluster development, the government plays a leading role to promote the development of the cluster, through knowledge spillover between clusters. After the industrial clusters enter the development stage, their innovation-driven capability and outward linkages increase, and regional innovation networks gradually take shape. The government no longer intervenes too much in a coercive manner, but plays more of a guiding role to promote competition and cooperation within the clusters, as well as providing support and guaranteeing the development of the clusters. As industrial clusters enter the transformation stage, the synergy mechanism within the cluster is able to function better, leading to the achievement of new breakthroughs and developments in the global division of production, while the government gives full play to its service role, promotes the optimization of the role of the synergy mechanism, plays a positive incentive role, provides more effective public services for the cluster, and helps the cluster to innovate and develop. The pharmaceutical industrial cluster innovation development process is shown in Figure 5.

## 5. Conclusions

Facing major restructuring of the global industry and the important transition period of innovation-driven development of the pharmaceutical industry, how to sustain innovation and development of pharmaceutical industrial clusters, in order to enhance international competitiveness, still requires systematic analysis and thought. Therefore, in this study, we analyzed the transformation and upgrading process of traditional pharmaceutical industrial clusters through a longitudinal case study and an in-depth analysis of the process of innovation and development of pharmaceutical industrial clusters, and then, explored the path and inner mechanism of innovative development for pharmaceutical industrial clusters. This study combined the development characteristics of pharmaceutical industrial clusters, filled the shortcomings of the literature regarding the evolutionary paths of traditional pharmaceutical industrial clusters and their mechanisms, and explored the three basic mechanisms of innovative development for traditional pharmaceutical industrial clusters. The main theoretical contributions of this study are as follows: first, the stages of pharmaceutical industrial cluster development were divided according to the different roles of government, thus updating the method of cluster stage division, and more comprehensively delving into the inner law of innovative development for pharmaceutical industrial clusters. Second, we further clarified the innovation evolutionary path and mechanism of traditional pharmaceutical industrial clusters, providing meaningful insights for a more reasonable and comprehensive interpretation of pharmaceutical industrial life cycle theory. Third, the logic of regional industry–academia–research cooperation and knowledge spillover was further clarified, and the transformation and upgrading model of the pharmaceutical industrial cluster was proposed to be guided by the government, cooperated and innovated by enterprises, and integrated into the global competition system.

Through the empirical analysis of the Shijiazhuang pharmaceutical excipients and preparations industrial cluster, it was found that the innovative development of traditional pharmaceutical industrial clusters is driven by many factors, and is subject to the interactions between three mechanisms. Among them, the self-organization mechanism comprises the necessary condition for pharmaceutical industrial clusters to realize the change from disorder to order, the innovation-driven mechanism provides the core driving force for the transformation and upgrading of pharmaceutical industrial clusters, and the outward associated mechanism is the main way by which pharmaceutical industrial clusters get rid of the closed country problem, and open external channels. The coordinated operation of the three mechanisms is a prerequisite for the innovative development of pharmaceutical industrial clusters to a higher stage. The key to the transformation of traditional pharmaceutical industrial clusters into innovative clusters relies on the construction of appropriate synergistic mechanisms at different stages of development, as well as the government’s role in promoting the cluster’s continuous innovation and development by “taking advantage of the situation”.

According to the above conclusions, combined with the existing problems, we put forward the following recommendations regarding the innovation development of pharmaceutical industrial clusters. On the one hand, under the combined action of demand growth, technological innovation, policy encouragement, talent gathering, and capital input, the pharmaceutical industry is expected to show a pattern of scale expansion, innovation upgrading, and competition differentiation for some time in the future. Pharmaceutical industrial clusters should improve their independent innovation capabilities, and take initiative to embed in the global value chain [56]. They should focus on the introduction of talent, enhancing their ability to cooperate with industry, academia, and research, and further strengthening the international division of labor and cooperation. At the same time, the risk investment and financing mechanism within the cluster should be improved, diversified channels of funding sources should be opened up, and the continuous innovation and development of the pharmaceutical industry cluster should be promoted.

On the other hand, in the process of pharmaceutical cluster development, the government should formulate policies that are appropriate to the time [57]. The macro-control of the government is a very important part of the development of the market economy; therefore, it is very important for the transformation and upgrading of the pharmaceutical industrial clusters that corresponding policies be formulated according to the different stages of cluster development [58]. The government should give full play to the platform role, in order to encourage government–industry–university–research collaborative innovation. The government should strengthen financial support for the internal science and technology innovation institutions of enterprises, research institutes, and universities, thus driving more enterprises, research institutes, and universities to carry out industry–university–research cooperation. Furthermore, regional economic development should be promoted by establishing a common sharing mechanism, which makes the advantages between subjects complementary and mutually beneficial.

## Figures and Tables

**Table 1:** Data collection and data composition.

Data Type	Description	Collection Methods
Access to Online Information	Websites, news, newspapers, etc.	Online and offline multi-channel collection
Interviews and Field Observations	Cluster management committee, leading enterprises	Multiple rounds of interviews and phone calls
File Information	Internal brochures, meeting minutes, official documents, etc.	Management committee and company
Database Retrieval Information	Domestic and foreign Journal Databases	Multi-person multi-dimensional search

**Table 2:** Pharmaceutical industrial cluster start-up stage interview codes.

Main Categories	Level of Coding	Secondary Coding	Initial Data Citation
Self-Organization	Resource Endowment	Labor Resources	*Wartime production (e.g., gauze, cotton, some alcohol); the production processes are very simple; there is simple technology and sufficient manpower.*
		Abundant Land Resources	*When the national development zone was first established, as there was a historical foundation and abundant land resources here.*
	Social Elements	Location Advantage	*Shijiazhuang is a location straddling semi-arid and semi-humid zones, offering unique conditions for the cultivation of medicinal herbs, production of raw materials, and research and development of pharmaceutical products.*
		Political Elements	*The predecessor of CSPC (ShiYao) was a pharmaceutical factory established in the base area of North China at that time. After the founding of the country, the major national projects of the First Five-Year Plan period included North China Pharmaceutical, and then, gradually concentrated on the construction of the factory.*
	Mutation factors	Historical path dependence	*The operation began in 1955. It was desirable to build a pharmaceutical factory in China, in order to have their own production at the earliest point possible. This matter was considered during the founding of new China, so it was built here.*
Innovation-Driven	Experiential Learning	Professionalism	*The drugs produced included a class of penicillin, a class of cephalosporin antibiotics. Such drugs are allergenic, so the production plant can only carry out the production of a single product. Thus, considering the cost problem, general pharmaceutical companies will produce the same class of drugs.*
		Heterogeneity	*Western medicine technology in developed countries is more advanced, but China also needs to retain its own advantages. YiLing Pharmaceutical and Shineway Pharmaceutical are part of the development of the heritage of traditional Chinese medicine.*
	Innovation Policy	Development Zone Construction	*In 1991, the State Council issued a document approving the establishment of Shijiazhuang National Hi-Tech Industrial Development Zone. In 1992, the People’s Government of Hebei Province approved the establishment of Shijiazhuang Economic and Technological Development Zone.*
		Investment Promotion	*The phase was started by spending a large amount of money to attract investment, encouraging companies to settle, bringing new innovations, and allowing companies to cluster.*
Outward Associated	Cluster Interaction	Technology Introduction and Collaboration	*At the beginning of the start-up, it was all about the production and processing of API, and the introduction of other people’s technologies; these are, of course, general production technologies and not core technologies*.
		Geographical Proximity	*The gap between the fast-developing clusters in Beijing and Tianjin (which are closer) is larger, but interaction between clusters can be encouraged to give full play to the positive externalities of quality resources.*

**Table 3:** Pharmaceutical industrial cluster development stage interview codes.

Main Categories	Level of Coding	Secondary Coding	Initial Data Citation
Self-Organization	Corporate Diversity	Technology-based SMEs	*In this period, there were 70 to 80 large enterprises, and the total business comprised more than 500 small enterprises. Many science- and technology-based small- and medium-sized enterprises gradually emerged.*
		Leading Enterprises	*The core of the development area consisted of three large plants—CSPC (ShiYao), NCPC (HuaYao), and Shijiazhuang No.4 pharmaceutical—which were pillar biopharmaceutical enterprises driving the development of the entire cluster.*
	Social Elements	Group Elements	*Geographical proximity has led to the formation of simple small groups, or simple partnerships, between some companies. It is up to the senior companies to allow some low-end enterprises to produce APIs* (*Active Pharmaceutical Ingredients), followed by their own processing into finished drugs.*
		Political Elements	*The provincial government has proposed four development goals for the development zone, in addition to an overall goal for a development zone.*
Innovation-Driven	The prototype of government–industry–academia	Cooperation with Universities	*Some new products or new technologies are to be developed in cooperation with universities.*
		Government Support	*For pharmaceutical companies, national policies have a great impact.*
		Corporate Connections	*Relatively speaking, there is a division of labor in which large companies relegate some technologies that they think may be less profitable to other companies.*
	Innovation Network	Platform Construction	*Establishing cluster platforms for financing and cooperation, supporting and leading the development of enterprises within the cluster, and managing large and small affairs at the cluster level.*
		Technology Breakthrough	*There are two aspects of technology: one is the tangible technology itself, and the other is its value. Depending on the quality of the product, the technology, value, and intellectual property will be more valuable and more meaningful.*
	R & D investment	Material input	*The main investments, such as those into research and development and advertising, are essential. The general investment costs account for about five percent of their production value, and two or three percent of their R & D funds. Advertising is essential, and the growth of the intangible assets of a company is dependent on advertising.*
		Human Resources	*The development zone to introduce incentives for the introduction of high-tech talent, for these technology-based demonstration enterprises, the introduction of support for scientific and technological innovation, and technology-based small- and medium-sized enterprises all require support policies. Every year, five percent of financial expenditures are used for incentives or support for scientific and technological innovation.*
Outward Associated	Investment channels	Financial Support	*With the continuous development of the company itself, the source of capital is varied. Listed companies have more diversified sources of capital.*
	Supply Chain	International Market Development	*Some companies export more, whereas some export less. CSPC (ShiYao) and NCPC (HuaYao) both have exports. Further business investment is also in the scope; that is to say, an enterprise requires some downstream enterprises in order to lead other teams, as well as investing in building factories.*
		Upstream and downstream bidding	*Internationalization is in line with the international situation, but also needs to be in line with the domestic situation. Companies also bid domestically, including their upstream and downstream products. There is a procurement and collection center, which has two bids per year to determine downstream suppliers, based on cost, quality, and supply capability.*

**Table 4:** Pharmaceutical industrial cluster innovation stage interview codes.

Main Categories	Level of Coding	Secondary Coding	Initial Data Citation
Self-Organization	Industry Chain	Cluster Learning	*The clustering of enterprises facilitates centralized and unified management, and is also conducive to the transfer of knowledge, and the formation of a complete industrial chain.*
		Leading Enterprises	*The leading company cooperates with chemical plants in Shijiazhuang and Zhao Counties for the supply of raw materials, due to the close distance and lower transportation costs. There is close cooperation in this regard.*
	Competing and Coexisting	Unified Cluster Development	*The Shijiazhuang pharmaceutical excipients and preparations industrial cluster mainly comprises five different functional clusters; that is, five functional areas with their own advantages and scientific and technological resources, where the functional differences make each area more specialized.*
		Price and Resource competition	*There is some competition, in terms of price and resources, among the small pharmaceutical companies in the cluster. However, due to the severe environmental problems occurring in the past two years, the degree of cooperation among the Chinese medicine factories has deepened, especially in terms of breakthroughs addressing some technical difficulties.*
Innovation-Driven	Subject interaction	Cooperation with Universities	*Some new products or new technologies are to be developed in cooperation with universities in Beijing and Tianjin.*
		Industrial Associations	*There are industrial associations and industrial alliances, and agreements have been signed between the management committee and these parties to jointly work on related projects, and build a platform for the enterprises to communicate and cooperate with each other.*
		Engineering Lab	*A gradual increase in the number of science and technology R & D institutions in the cluster is witnessed. There is a national testing center, a provincial technical center, a post-doctoral station, and several engineering laboratories.*
	Technology Innovation	Intellectual Property	*Each of the cluster’s pharmaceutical companies has hundreds of patents. They attach great importance to intellectual property rights, and include research institutes and research laboratories.*
		R & D Team	*A strategic cooperation alliance with Hebei University has been previously established, and now there is some cooperation with the China University of Medicine, Hebei University of Economics and Business, and Hebei University of Engineering.*
	Innovation Environment	Talent Introduction	*A number of academic workstations and post-doctoral stations in the cluster have attracted high-level talent, creating a permanent “think tank” for the cluster. Based on a high-level R & D team, in-depth research continues, and the development and transformation of innovative results is accelerated. The cluster includes many famous scholars and technical backbones from home and abroad.*
		“Emptying the cage, changing the birds”	*Enterprises with backward production capacity are to be gradually eliminated, with the additional aim to incubate innovative subjects for enterprises that can be transformed and guided, as well as for those that will be newly settled in.*
Outward Associated	Global Value Chain	International Business	*Talent is very important—thus, there are preparations to facilitate the introduction of returnees, as well as to set up country industrial parks and a returnee business park. In addition, some international business development is carried out.*
		International Certification	*International certification is in progress. Some medicines have passed the EU certification, and some have passed the Japanese certification; furthermore, several powder injections have passed the EU certification.*
	Enhance international competitiveness	Open door to the outside world	*In recent years, the level of openness has continued to improve. Shijiazhuang Economic and Technological Development Zone foreign-invested enterprises and foreign trade export enterprises are increasing, and the amount of foreign investment and export earnings are also growing.*
		Overseas study	*In the field of scientific and technological innovation, the key laboratories established are in cooperation with research institutes, doctoral and academic workstations, national monitoring centers, and so on (i.e., R & D centers). CSPC (ShiYao) has established global R & D centers in the U.S. and other locations.*

## Data Availability

Shijiazhuang Statistical Yearbook: http://tjj.sjz.gov.cn/ (accessed on 7 January 2022).
